# Safety and Accuracy of Sentinel Lymph Node Biopsy Alone in Clinically Node-Positive Patients Undergoing Upfront Surgery for Invasive Breast Cancer: A Systematic Review

**DOI:** 10.3390/curroncol30030235

**Published:** 2023-03-07

**Authors:** Olivia Lovrics, Brendan Tao, Elena Parvez

**Affiliations:** 1 Department of Surgery, McMaster University, 1280 Main Street West, Hamilton, ON L8S 4K1, Canada; 2 Faculty of Medicine, University of British Columbia, 317-2194 Health Sciences Mall, Vancouver, BC V6T 1Z3, Canada; 3 Juravinski Hospital and Cancer Centre, 711 Concession Street, Hamilton, ON L8V 1C3, Canada

**Keywords:** de-escalation, axillary surgery, sentinel lymph node biopsy, axillary dissection, targeted axillary surgery

## Abstract

Landmark trials (Z0011 and AMAROS) have demonstrated that axillary lymph node dissection (ALND) can be safely omitted in patients with breast cancer and 1–2 positive sentinel nodes. Extrapolating from these and other cardinal studies such as NSABP B-04, guidelines state that patients with 1–2 needle biopsy-proven positive lymph nodes undergoing upfront surgery can have sentinel lymph node biopsy (SLNB) alone. The purpose of this study is to systematically review the literature to identify studies examining the direct application of SLNB in such patients. EMBASE and Ovid MEDLINE were searched from inception to 3 May 2022. Studies including patients with nodal involvement confirmed on pre-operative biopsy and undergoing SLNB were identified. Studies with neoadjuvant chemotherapy were excluded. Search resulted in 2518 records, of which 68 full-text studies were reviewed, ultimately yielding only 2 studies meeting inclusion criteria. Both studies used targeted axillary surgery (TAS) with pre-operative localization of the biopsy-proven positive node in addition to standard SLNB techniques. In a non-randomized single-center prospective study, Lee et al. report no regional recurrences in patients undergoing TAS or ALND, and no difference in distant recurrence or mortality at 5 years. In the prospective multicenter TAXIS trial by Webber et al., the median number of positive nodes retrieved with TAS in patients undergoing upfront surgery was 2 (1, 4 IQR). Within the subset of patients who underwent subsequent ALND, 61 (70.9%) had additional positive nodes, with 26 (30.2%) patients having ≥4 additional positive nodes. Our review demonstrates that there is limited direct evidence for SLNB alone in clinically node-positive patients undergoing upfront surgery. Available data suggest a high proportion of patients with residual disease in this setting. While the totality of the data, mostly indirect evidence, suggests SLNB alone may be safe, we call on clinicians and researchers to prospectively collect data on this patient population to better inform decision-making.

## 1. Introduction

The last half-century has seen a fundamental evolution in the surgical management of invasive breast cancer to reduce the morbidity of treatment. Much research has focused on the de-escalation of axillary surgery as axillary lymph node dissection (ALND) is associated with an estimated 25% risk of lymphedema, which can reduce the quality of life in affected patients [1,2,3]. The safety of sentinel lymph node biopsy(SLNB) in node-negative patients was established by the NSABP B-32 trial, which showed no difference in overall survival between patients undergoing ALND and SLNB [4]. Building on this, trials including IBCSG-23, Z0011, and AMAROS demonstrated no difference in risk of recurrence in patients with a limited number of sentinel nodes involved with micrometastatic or macrometastatic disease treated with SLNB alone compared to completion ALND, undergoing breast-conserving surgery or mastectomy [5,6,7]. However, patients with clinically node-positive disease are not included in these trials. For patients with clinically node-positive disease treated with neoadjuvant chemotherapy and found to have a complete clinical response to treatment, ACOSOG 1071, SN FNAC, and SENTINA demonstrated that there can be an acceptably low false negative rate of SLNB with the use of dual tracer technique, routine immunohistochemistry, and retrieval of a minimum of three nodes [8,9,10]. Reported rates of nodal complete response following neoadjuvant chemotherapy range from 35–63% meaning these patients can be spared an ALND [11]. However, while the results of the prospective randomized ALLIANCE A011202 trial (ClinicalTrials.gov Identifier: NCT01901094) are anticipated, guidelines currently recommend completion of ALND for residual nodal disease identified with SLNB following neoadjuvant chemotherapy [12]. Applied in this setting, SLNB following neoadjuvant chemotherapy in node-positive patients is a staging procedure, rather than a therapeutic procedure. In support of this current recommendation is a National Cancer Database Study which found worse 5-year overall survival in patients with node-positive disease undergoing neoadjuvant chemotherapy and treated with SLNB alone compared to ALND [13].

Management of the axilla has become even more complex with the results of the RxPONDER trial, which demonstrated that post-menopausal women with node-positive, hormone-receptor-positive, Her2-negative disease and an OncotypeDx Score^®^ ≤ 25 do not benefit from cytotoxic chemotherapy [14]. With the results of this trial, more women with clinically node-positive disease will undergo upfront surgery rather than neoadjuvant chemotherapy. Currently, the National Comprehensive Cancer Network (NCCN) guidelines indicate that women with 1–2 positive lymph nodes confirmed on pre-operative needle biopsy undergoing upfront surgery can be treated with sentinel lymph node biopsy alone [15]. This recommendation is extrapolated from the results of the above-described trials, as well the cardinal NSABP B-04 trial which demonstrated that even in patients with node-positive breast cancer, axillary dissection compared to no axillary surgery does not result in an improvement in overall survival [16]. Different then using SLNB in node-positive patients treated with neoadjuvant chemotherapy, SLNB in this setting is intended to remove a small burden of node-positive disease.

Notably, ALND is still recommended after SLNB in patients who are found to have >2 positive sentinel lymph nodes (SLN) or >2 positive nodes detected on imaging. However, physical exams and pre-operative imaging lack accuracy in assessing the extent of nodal disease [17,18]. The ability of axillary US to exclude ≥3 is limited, with a false negative rate of 30–37% [19]. The ability of MRI to differentiate ≥3 nodes is similarly limited, with a false negative rate of 15–55% [19]. Thus, clinicians are challenged with the difficulty of differentiating between two and three involved nodes and whether such patients undergoing upfront surgery are more appropriate for SLNB or ALND.

This review aims to identify and synthesize direct evidence assessing the accuracy and oncologic safety of SLNB alone for axillary surgical management in patients with pathologically confirmed positive nodes undergoing upfront surgery for invasive breast cancer. 

## 2. Methods

### 2.1. Study Design

This study describes the literature regarding the accuracy and oncologic safety of SLNB in breast cancer patients undergoing upfront surgery with positive nodes confirmed on pre-operative needle biopsy. The study was designed following the Preferred Reporting Items for Systematic Reviews and Meta-Analyses (PRISMA) [20]. The study was filed retrospectively with PROSPERO [CRD42023393449]. The search strategy (Appendix A) was prepared by the study authors and with the assistance of a medical librarian. EMBASE and Ovid MEDLINE were searched from the time of database inception to 3 May 2022. Major terms included breast neoplasm, node-positive, and sentinel lymph node biopsy. Similar terms and explosion features were used. Reference lists were hand-searched for additional relevant articles.

### 2.2. Study Eligibility and Selection

Inclusion criteria were primary studies investigating female patients with invasive breast cancer and with clinically node-positive breast cancer undergoing upfront SLNB with reporting of outcome data (e.g., technical feasibility of SLNB in this setting, or recurrence, and survival). Node positive was defined as nodal involvement confirmed on pre-operative biopsy. Exclusion criteria were studies with non-human or male subjects, non-invasive breast cancer, lack of pathological confirmation of nodal status, prior neoadjuvant chemotherapy, no SLNB, and/or no data regarding outcomes. Studies including patients treated with neoadjuvant chemotherapy were excluded as current guidelines recommend ALND for residual disease. All primary studies, including published abstracts and case series studies, were eligible. Case reports and reviews were excluded. Studies were not excluded based on language of publication. 

### 2.3. Data Extraction 

Screening of studies was completed using Covidence Systematic Review Software (Melbourne, Australia) [21]. After deduplication, three independent screeners reviewed studies using standardized criteria; each study was screened by two reviewers and disagreements were settled by consensus. Full-texts of studies that were recommended for inclusion were retrieved and underwent full-text review. Full texts were assessed independently and in duplicate by three reviewers, with reasons documented for study exclusions. Data collection was completed using a standardized extraction form by 2 reviewers. Limited identified data precluded statistical analysis of collected data. A narrative summary of findings from the available literature is presented. 

## 3. Results

**Figure 1:**
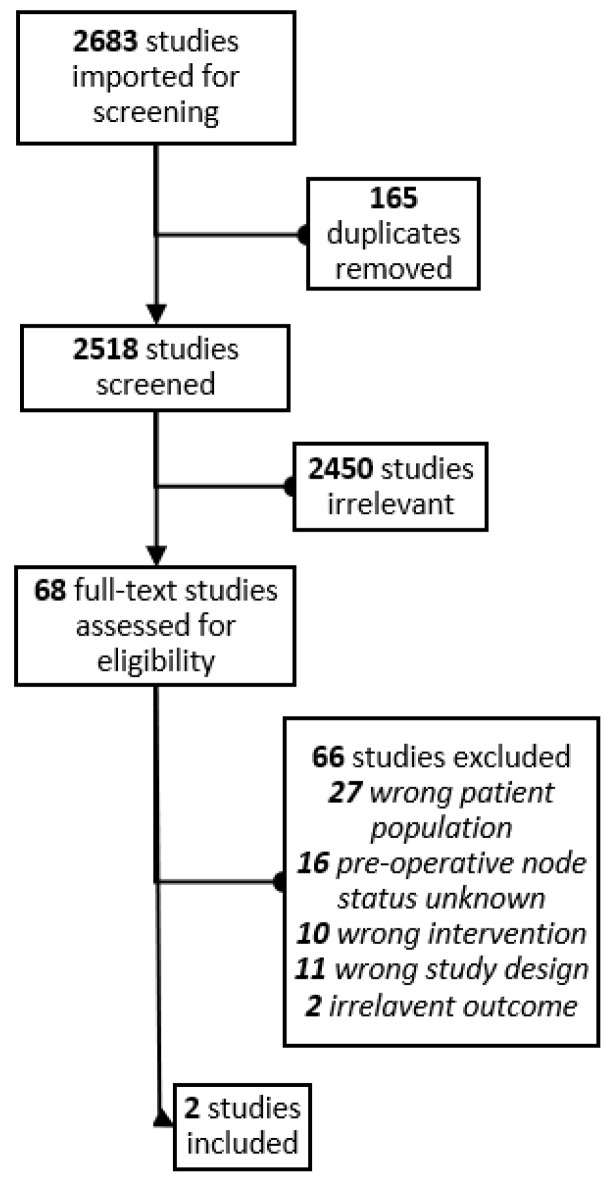
PRISMA diagram.

A total of 2683 citations with 165 duplicate records were identified, resulting in 2518 studies undergoing title and abstract screening. Full text review was conducted on 68 studies, ultimately yielding 2 studies for inclusion: Lee et al. (non-randomized prospective study) [19] and Weber et al. (prospective multicenter sub-study of the TAXIS trial) [22]. The PRISMA flow diagram is presented in Figure 1. Study features, patient characteristics, tumor details, and study outcomes are presented in Table 1. Both studies used a form of targeted or tailored axillary surgery technique to mark the positive nodes in addition to the standard SLNB technique. The method of targeted axillary sampling (TAS) used in the study by Lee et al. involved preoperative image-guided tattooing of positive lymph nodes using a charcoal solution and removal of these nodes and sentinel nodes, while not exposing axilla landmarks including the long thoracic nerve, thoracodorsal nerve, and axillary vein, and pathologically if 5–10 nodes were removed [19]. Tailored axillary surgery was defined by Weber et al. as the removal of pre-operative marked nodes, intra-operatively palpable nodes in addition to the sentinel lymph nodes [22]. Herein, both will be referred to as TAS.

The study by Lee et al. included patients from a single institution in South Korea and compared those undergoing TAS (*n* = 65) versus conventional ALND (*n* = 64) [19]. In this non-randomized prospective study, patients underwent TAS if they refused conventional ALND. Statistically significant differences did not exist between groups in age, pathologic stage, and biomarker profile. The TAS technique used was an injection of a charcoal solution into biopsy-proven positive nodes prior to surgery, in addition to the standard SLNB technique, and without dissection of the axillary vein, thoracodorsal bundle, and long thoracic nerve. Patients were excluded from the TAS arm if they had ≤4 LNs removed and excluded from the ALND arm if they had ≤10. The number of patients excluded was not reported. The follow-up period was mean 65.7 months in those undergoing TAS and 73.1 months in those undergoing ALND. There was no significant difference in the mean number of retrieved positive nodes between ALND and TAS (1.6 vs. 1.6, *p* = 0.772), though significantly more nodes were retrieved overall in the ALND group (19.6 vs. 7.7, *p* < 0.001). The success of retrieving the pre-operative marked node was not reported. None of the patients undergoing ALND received adjuvant radiation. Patents undergoing TAS were randomly assigned to receive adjuvant nodal radiation, though the method of randomization was not described. There were 39 (60%) patients in the TAS cohort who received adjuvant radiation. They report no nodal recurrences in either arm and no difference in local recurrence (ALND: 1.6%; TAS: 1.5%), distant recurrence (ALND: 4.7%; TAS: 6.2%), and mortality (ALND: 1.6%; TAS: 3.1%) during over 5-year follow-up, in this small non-randomized study. Rates of post-operative complications such as lymphedema were not reported. 

The study by Weber et al. is a pre-specified sub-study to assess surgical outcomes of the TAXIS trial [22]. The TAXIS trial is a prospective clinical trial in which patients with node-positive disease will undergo TAS and then be randomized to ALND or no ALND and regional radiation based on the extent of surgery (ClinicalTrials.gov Identifier: NCT03513614). The study included patients from multiple institutions across Europe and included patients with node-positive disease undergoing both neoadjuvant chemotherapy and upfront surgery. Various methods for marking the clipped positive node were used including a magnetic and radioactive seed, wire, and tattoo. There were 166 patients with node-positive disease undergoing upfront TAS included in the study, of which 81 had abnormal nodes seen on imaging only, and 85 had palpable abnormal nodes. Weber et al. report that TAS was associated with a false negative rate of 2.4%, with the retrieval of the clipped node in 96.4% of cases, and a diagnostic accuracy rate of 97.6% [22]. The final pathologic staging was pN1 in 102 (61.4%) patients. In patients who underwent subsequent ALND (*n* = 86), a median of 14 (IQR 9–18) additional nodes were removed, of which a median 2 (IQR 0–6) were positive. Only 25 (29.1%) had no additional positive nodes found at ALND. There were 26 (30.2%) patients who had ≥4 positive nodes identified. 

## 4. Discussion

Complications associated with axillary lymph node dissection include lymphedema, chronic pain, paresthesias, and shoulder immobility [23]. Lymphedema can impact physical, psychological, and social quality of life. Given the morbidity of ALND, research efforts into the de-escalation of axillary management in breast cancer are ongoing. Sentinel lymph node biopsy has been adopted as the standard of care in patients with node-negative breast cancer. Large, randomized trials have demonstrated that patients with a limited burden of positive sentinel nodes can be spared ALND. While consensus guidelines now allow for the use of SLNB alone in patients with pre-operative biopsy-proven node-positive disease and 1–2 abnormal lymph nodes on imaging undergoing upfront surgery, our review highlights the striking lack of data on SLNB alone in this patient population. Only 2 studies meeting inclusion criteria with data on 231 patients were identified through a systematic review of the literature. Given the substantive amount of work completed addressing this same question in the neoadjuvant setting, the lack of data for patients undergoing upfront surgery is truly striking. This is especially the case given the results of RxPONDER which demonstrate that select patients with node-positive disease can be spared chemotherapy [14].

The studies included in this review used different outcome metrics which precluded the ability to complete statistical analyses. Both studies used targeted axillary surgery techniques where the positive node was clipped/marked, in addition to standard sentinel lymph node biopsy. Clipping of the biopsy-proven positive node and subsequent retrieval has been shown to decrease the false negative rate of SLNB in node-positive patients following neoadjuvant chemotherapy [24,25]. However, the added benefit of clipping the node over standard SLNB on the long-term oncologic outcomes has been questioned [3]. Logically, this technique can be extrapolated to the use of SLNB in the upfront surgery setting to ensure retrieval of the known positive node. In the studies included in this systematic review, the mean number of retrieved nodes was 7.7 in the study by Lee et al., and the median number of nodes retrieved 5 was in the study by Weber et al., significantly more than are removed during standard SLNB [26]. The study by Weber et al. is a pre-planned feasibility sub-study of a prospective, multicenter, randomized trial, where long-term oncologic outcomes will be assessed. In this feasibility sub-study, the false-negative rate of TAS was only 2.4% and retrieval of the clipped node was successful in 96.4% of cases. 

In the study by Weber et al., most patients randomized to subsequent ALND were found to have residual disease in the axilla (70.9%). The rate of residual disease in this study was significantly higher than the burden of residual disease in the ALND arm of the Z0011 trial (27%) [7,27], raising the question of whether the same criteria for completion of ALND should be applied. The NCCN guidelines allowing SLNB in patients with 1–2 needle biopsy-proven positive nodes recommend that those with ≥2 positive nodes have ALND. Following these guidelines, the vast majority of patients would require ALND. There is currently insufficient data to determine whether this is necessary. The implication of this residual disease, especially in the era of modern multi-modality treatment, in patients undergoing omission of ALND on long-term oncologic outcomes will not be known until 2029 when results of the phase III trial are expected. 

The risk of bias was high in one study (Lee et al.), where the number of patients who were excluded due to an inadequate number of nodes retrieved was not reported [19]. Nonetheless, this study was unique in that it reported long-term oncologic outcomes and in this small sample size of 65 patients with node-positive disease who underwent limited axillary surgery, there were no axillary recurrences, and there was no difference in distant metastases or death when compared to patients undergoing ALND. 

An ongoing Canadian study, Targeted Axillary Dissection in Early Stage Node-Positive Breast Cancer (TADEN) trial (ClinicalTrials.gov Identifier: NCT04671511) will measure the success rate and accuracy of SLNB with clipping and removing the biopsy-proven node using radioactive seed localization (RSL) in patients with biopsy-proven positive nodes. Completion node dissection is recommended in this study if the clipped positive node is not retrieved, if four nodes or more are positive, or if three nodes are positive in the absence of axillary radiation. This study will assess if there is a reduction in recommendations for the completion of ALND. 

Finally, as the COVID-19 pandemic continues to affect the over-burdened healthcare system, the use of SLNB in node-positive patients can be considered in view of the state of the system as well. The safe omission of ALND in clinically node-positive patients undergoing upfront surgery presents an opportunity for potential healthcare cost saving due to decreased incidence of morbidity of ALND. However, it should be noted, that potentially more patients may have two surgeries with this approach (SLNB followed by ALND, compared to ALND alone). Additionally, depending on the method of localization used for targeted axillary surgeries, the costs of this procedure tend to exceed that of SLNB alone [3]. As healthcare systems around the world work to increase capacity to address backlogs in breast cancer screening and treatment, resources focused on the cost-effective yet safe assessment of symptomatic women should be paramount to reduce resultant morbidity and mortality [28].

## 5. Conclusions

There is certainly a group of patients with pre-operative needle biopsy-proven node-positive disease undergoing upfront surgery who can be spared an ALND. However, this review demonstrates a current lack of substantial direct evidence supporting the application of SLNB in patients with biopsy-proven node-positive disease undergoing upfront surgery. While the totality of the data, mostly indirect evidence, suggests this may be a safe approach, more data are needed. This is a call for clinicians and researchers to prospectively collect data on this patient population to better understand the proportion of patients undergoing subsequent completion of ALND, long-term oncologic outcomes, and cost-effectiveness, which will ultimately help clinical decision-making.

## Figures and Tables

**Table 1:** Study characteristics and outcomes.

Authors	Year	Country/ Region	Study Design	Inclusion Criteria	Patients	Follow-Up	Outcomes
Lee et al. [19]	2019	South Korea	Single-center, non-randomized double arm prospective cohort study comparing TAS vs. ALND (treatment arm assigned based on patient preference	cT1-2N1 BC	*n* = 64 (ALND) *n* = 65 (TAS)	5-years	Mean number of nodes retrieved: 19.6 vs. 7.7 (ALND vs. TAS) Mean number of metastatic nodes: 1.6 vs. 1.6 (ALND vs. TAS) Local recurrence, n(%): 1 (1.6%) vs. 1 (1.5%) (ALND vs. TAS) Distant recurrence, n(%): 3 (4.7%) vs. 4 (6.2%) (ALND vs. TAS) Death, n(%): 1(1.6%) vs. 2 (3.1%) (ALND vs. TAS) No nodal recurrences in either arm
Weber et al. [22]	2021	Europe	International multicenter, prospective study embedded in a randomized trial (TAXIS) comparing TAS vs. TAS + ALND (feasibility study)	cT1-3 N1-2 BC, All breast cancer subtypes	*n* = 166 treated with upfront surgery with TAS, of which 86 underwent subsequent ALND	Post-surgery	FNR for TAS: 2.4% Retrieval of clipped node: 96.4% Median # of nodes(IQR) retrieved with TAS: 5 (3, 7) Median # of additional nodes (IQR) retrieved ALND: 14 (9, 18) Median # of positive nodes (IQR) with TAS: 2 (1, 4) Median # of additional positive nodes with ALND: 2 (0, 6) Number of patients with additional positive nodes removed with ALND (n, %): 61 (70.9%) - 1 additional positive node: 17 (29.1%) - 2 additional positive nodes: 6 (7.0%) - 3 additional positive nodes: 6 (7.0%) - 4 additional positive nodes: 6 (7.0%) - >4 additional positive nodes: 26 (30.2%) (Appendix A)

Abbreviations: TAS = targeted axillary sampling, ALND: axillary lymph node dissection, FNR: false negative rate, IQR: interquartile range.

## Data Availability

Upon request.

## Appendix A. Search Strategy

**Table:**

Embase Strategy	Ovid MEDLINE Strategy
breast neoplasm.mp. or Breast Neoplasms/ ((mammar* or breast$ or lobular*) adj3 (mass$ or cancer$ or tumo?r$ or neoplasm$ or carcinoma$ or adenocarcinoma$)).mp. 1 or 2 ((clinical$ adj3 (node positive or lymph node positive or sentinel lymph node positive)) or sentinel lymph node).mp. (clinical* node positive or cN+ or N+).mp. 4 or 5 (sentinel lymph node biopsy or SNLB or SNB or lymph node biopsy or lymph node sampl$ or (recurrence and sentinel lymph node)).mp. 3 and 6 and 7 ((clinical$ adj3 (node negative or lymph node negative or sentinel lymph node negative)) or (node negative or lymph node negative or sentinel lymph node negative)).mp. case report?.mp. case series.mp. normal human/ human experiment/ 8 not (9 or 10 or 11 or 12 or 13) limit 14 to (human and article) prediction.mp. neoadjuvant.mp. in vitro.mp. 15 not (16 or 17 or 18) dissection.mp. 19 and 20	Breast neoplasm.mp. or Breast Neoplasms/ ((mammar* or breast$ or lobular*) adj3 (mass$ or cancer$ or tumo?r$ or neoplasm$ or carcinoma$ or adenocarcinoma$)).mp. 1 or 2 (clinical$ adj3 (node positive or lymph node positive or sentinel lymph node positive)).mp. (clinical* node positive or cN+ or cN0+ or N+).mp. 4 or 5 (sentinel lymph node biopsy or SNLB or SNB or lymph node biopsy or lymph node sampl$).mp. 3 and 6 and 7

## References

[1] Naoum G.E., Roberts S., Brunelle C.L., Shui A.M., Salama L., Daniell K., Gillespie T., Bucci L., Smith B.L., Ho A.Y. (2020). Quantifying the impact of axillary surgery and nodal irradiation on breast cancer-related lymphedema and local tumor control: Long-term results from a prospective screening trial. J. Clin. Oncol..

[2] McEvoy M.P., Gomberawalla A., Smith M., Boccardo F.M., Holmes D., Djohan R., Thiruchelvam P., Klimberg S., Dietz J., Feldman S. (2022). The prevention and treatment of breast cancer—related lymphedema: A review. Front. Oncol..

[3] Galimberti V., Ribeiro Fontana S.K., Vicini E., Morigi C., Sargenti M., Corso G., Magnoni F., Intra M., Veronesi P. (2023). “This house believes that: Sentinel node biopsy alone is better than TAD after NACT for cN+ patients”. Breast.

[4] Krag D.N., Anderson S.J., Julian T.B., Brown A.M., Harlow S.P., Costantino J.P., Ashikaga T., Weaver D.L., Mamounas E.P., Jalovec L.M. (2010). Sentinel-lymph-node resection compared with conventional axillary-lymph-node dissection in clinically node-negative patients with breast cancer: Overall survival findings from the NSABP B-32 randomised phase 3 trial. Lancet Oncol..

[5] Miranda K., Pace D., Cintron R., Rodrigues J.C.F., Fang J., Smith A., Rohloff P., Coelho E., De Haas F., Souza D. (2011). IBCSG 23-01 randomised controlled trial comparing axillary dissection versus no axillary dissection in patients with sentinel node micrometastases. Lancet Oncol..

[6] Donker M., van Tienhoven G., Straver M.E., Meijnen P., van de Velde C.J.H., Mansel R.E., Cataliotti L., Westenberg A.H., Klinkenbijl J.H.G., Orzalesi L. (2014). Radiotherapy or surgery of the axilla after a positive sentinel node in breast cancer (EORTC 10981-22023 AMAROS): A randomised, multicentre, open-label, phase 3 non-inferiority trial. Lancet Oncol..

[7] Giuliano A., Hunt K.K., Ballman K.V., Beitsch P.D., Whitworth P.W., Blumencranz P.W., Leitch A.M., Mccall L.M., Morrow M. (2011). Axillary Dissection vs No Axillary Dissection in Women With Invasive Breast Cancer. JAMA.

[8] Kuehn T., Bauerfeind I., Fehm T., Fleige B., Hausschild M., Helms G., Lebeau A., Liedtke C., von Minckwitz G., Nekljudova V. (2013). Sentinel-lymph-node biopsy in patients with breast cancer before and after neoadjuvant chemotherapy (SENTINA): A prospective, multicentre cohort study. Lancet Oncol..

[9] Boileau J.F., Poirier B., Basik M., Holloway C.M.B., Gaboury L., Sideris L., Meterissian S., Arnaout A., Brackstone M., McCready D.R. (2015). Sentinel node biopsy after neoadjuvant chemotherapy in biopsy-proven node-positive breast cancer: The SN FNAC study. J. Clin. Oncol..

[10] Boughey J.C., Suman V.J., Mittendorf E.A., Ahrendt G.M., Wilke L.G., Taback B., Leitch A.M., Kuerer H.M., Bowling M., Flippo-Morton T.S. (2013). Sentinel lymph node surgery after neoadjuvant chemotherapy in patients with node-positive breast cancer: The ACOSOG Z1071 (alliance) clinical trial. JAMA.

[11] Pilewskie M., Morrow M. (2017). Axillary nodal management following neoadjuvant chemotherapy: A review. JAMA Oncol..

[12] Maggi N., Nussbaumer R., Holzer L., Weber W.P. (2022). Axillary surgery in node-positive breast cancer. Breast.

[13] Almahariq M.F., Levitin R., Quinn T.J., Chen P.Y., Dekhne N., Kiran S., Desai A., Benitez P., Jawad M.S., Gustafson G.S. (2021). Omission of Axillary Lymph Node Dissection is Associated with Inferior Survival in Breast Cancer Patients with Residual N1 Nodal Disease Following Neoadjuvant Chemotherapy. Ann. Surg. Oncol..

[14] Kalinsky K., Barlow W.E., Gralow J.R., Meric-Bernstam F., Albain K.S., Hayes D.F., Lin N.U., Perez E.A., Goldstein L.J., Chia S.K.L. (2021). 21-Gene Assay to Inform Chemotherapy Benefit in Node-Positive Breast Cancer. N. Engl. J. Med..

[15] Breast Cancer Version 4 (2022). NCCN Clinical Practice Guidelines in Oncology. Published 2022..

[16] Fisher B., Montague E., Redmon C., Barton B., Borland D., Fisher E., Deutsch M., Schwarz G., Margolese R., Donegan W. (1977). Comparison of radical mastectomy with alternative treatments for primary breast cancer: A first report of results from a prospective rnadomized clinical trial. Cancer.

[17] Riedel F., Schaefgen B., Sinn H.P., Feisst M., Hennigs A., Hug S., Binnig A., Gomez C., Harcos A., Stieber A. (2021). Diagnostic accuracy of axillary staging by ultrasound in early breast cancer patients. Eur. J. Radiol..

[18] Chang J.M., Leung J.W.T., Moy L., Ha S.M., Moon W.K. (2020). Axillary nodal evaluation in breast cancer: State of the art. Radiology.

[19] Lee J., Jung J.H., Kim W.W., Lee R.K., Kim H.J., Kim W.H., Park J.Y., Jeong J.Y., Chae Y.S., Lee S.J. (2019). 5-year oncological outcomes of targeted axillary sampling in pT1-2N1 breast cancer. Asian J. Surg..

[20] Moher D., Liberati A., Tetzlaff J., Altman D.G., the PRISMA Group (2009). Preferred reporting items for systematic reviews and meta-analyses: The PRISMA statement. BMJ.

[21] Covidence systematic review software. http://www.covidence.org.

[22] Weber W.P., Matrai Z., Hayoz S., Tausch C., Henke G., Zwahlen D.R., Gruber G., Zimmermann F., Seiler S., Maddox C. (2021). Tailored axillary surgery in patients with clinically node-positive breast cancer: Pre-planned feasibility substudy of TAXIS (OPBC-03, SAKK 23/16, IBCSG 57-18, ABCSG-53, GBG 101). Breast.

[23] Zhang J.Q., Montagna G., Sevilimedu V., Abbate K., Charyn J., Mehrara B., Morrow M., Barrio A.V. (2022). Longitudinal Prospective Evaluation of Quality of Life After Axillary Lymph Node Dissection. Ann. Surg. Oncol..

[24] Caudle A.S., Yang W.T., Krishnamurthy S., Mittendorf E.A., Black D.M., Gilcrease M.Z., Bedrosian I., Hobbs B.P., Desnyder S.M., Hwang R.F. (2016). Improved axillary evaluation following neoadjuvant therapy for patientswith node-positive breast cancer using selective evaluation of clipped nodes: Implementation of targeted axillary dissection. J. Clin. Oncol..

[25] Kuemmel S., Heil J., Rueland A., Seiberling C., Harrach H., Schindowski D., Lubitz J., Hellerhoff K., Ankel C., Graßhoff S.T. (2022). A Prospective, Multicenter Registry Study to Evaluate the Clinical Feasibility of Targeted Axillary Dissection (TAD) in Node-positive Breast Cancer Patients. Ann. Surg..

[26] Dixon J.M., Grewar J., Twelves D., Graham A., Martinez-Perez C., Turnbull A. (2020). Factors affecting the number of sentinel lymph nodes removed in patients having surgery for breast cancer. Breast Cancer Res. Treat..

[27] Giuliano A.E., Mccall L., Beitsch P., Whitworth P.W., Blumencranz P., Leitch A.M., Saha S., Hunt K.K., Morrow M., Ballman K. (2010). Locoregional Recurrence after Sentinel Lymph Node Dissection with or without Axillary Dissection in Patients with Sentinel Lymph Node Metastases: The American College of Surgeons Oncology Group Z0011 Randomized Trial. Ann. Surg..

[28] Alagoz O., Lowry K.P., Kurian A.W., Mandelblatt J.S., Ergun M.A., Huang H., Lee S.J., Schechter C.B., Tosteson A.N.A., Miglioretti D.L. (2021). Impact of the COVID-19 Pandemic on Breast Cancer Mortality in the US: Estimates From Collaborative Simulation Modeling. J. Natl. Cancer Inst..

